# The Research Objective Structured Clinical Exam (R-OSCE): An Innovative Tool to Assess Clinical and Translational Research Competencies

**DOI:** 10.15694/mep.2021.000143.1

**Published:** 2021-05-24

**Authors:** Phillip A. Ianni, Brenda L. Eakin, Elias M. Samuels, Ellen Champagne, Vicki L. Ellingrod

**Affiliations:** 1 University of Michigan

**Keywords:** OSCE, Clinical Research, Translational Research, Competency, Education, Assessment

## Abstract

This article was migrated. The article was marked as recommended.

Introduction

The Research Objective Structured Clinical Exam (R-OSCE) described in this paper was designed as part of a comprehensive program to assess competency in specific domains of clinical and translational research (CTR) for students enrolled in a 12-week introductory summer research program.

Methods

The program curriculum was mapped to core competencies developed by the National Center for Translational Science (NCATS) and used to develop R-OSCE stations. Twelve stations were developed, with five administered during orientation as a practice test and seven administered post-program. A scoring rubric using an anchored scale of 1-5 was developed and six qualified raters were trained in its use. The exam was self-paced and delivered through a secure online computer-based platform.

Results

Forty-seven students (three cohorts) completed the post-program R-OSCE. Most respondents scored at a score of 3 (developing competence) or higher on most stations for both the practice and post-program exams, the exceptions being the stations involving writing research questions and engaging communities in research. Students indicated they liked demonstrating CTR skills through the R-OSCE. Most participants agreed that exam tasks were related to stated program competencies and that stations were realistic.

Discussion

The R-OSCE is best used as part of a comprehensive assessment program and may be useful in providing formative feedback to trainees that they can share with their mentors. Additionally, this study demonstrated that it could feasibly be used to evaluate the effectiveness of research education programs. However, additional time was needed to train raters and score the R-OSCE. Modifications were made to administer the exam through use of an online format with a modest budget. The computer-based format provides a solution to the current need for assessments that can be administered remotely.

## Introduction

For more than a decade, the National Center for Advancing Translational Science (NCATS), which administers the Clinical and Translational Science Award (CTSA), has worked to define the knowledge, skills, and attitudes (also called competencies) that are considered critical to the conduct of clinical and translational research (CTR) and the training of the translational science workforce ( National Center for Advancing Translational Sciences, 2019). Developed collaboratively with a workgroup from the former CTSA Education and Career Development Key Function Committee, these competencies are organized into 14 core thematic areas that are analogous to competency domains ( Clinical & Translational Science Awards Program, 2019). Since their development, the CTR core competencies have been widely adopted by members of the CTSA Consortium to guide development of their training and education programs.

In recent years, competency-based curricula and portfolio-based assessments have been developed for graduate level CTR training, with limited empirical validation ( Dilmore, Moore and Bjork, 2013; Robinson *et al.*, 2015; Mayowski, Norman and Kapoor, 2018). For example, several self-efficacy assessments, such as the Clinical Research Appraisal Inventory (CRAI) and the Competency Index for Clinical Research Professionals (CICRP), have been developed for a range of CTR competencies ( Lipira *et al.*, 2010; Robinson *et al.*, 2013; Hornung *et al*., 2019). Self-assessments like these can be easy to administer ( Bray *et al.*, 2011) and are an integral part of practice-based learning ( Davis *et al.*, 2006). However, research has shown that self-assessment has the potential for errors in estimation of perceived competence ( Hodges, Regehr and Martin, 2001; Dunning, Heath and Suls, 2004; Davis *et al.*, 2006; Bray *et al.*, 2011). Therefore, there is a need to develop more objective assessments to better assess attainment of CTR competencies.

The Objective Structured Clinical Examination (OSCE), first described by Harden and colleagues ( Harden *et al.*, 1975), is a well-validated measure widely used in health professions education programs for competency assessment and as a component of the clinical certification and licensure process ( Rushforth, 2007; Albino *et al.*, 2008; Khan *et al.*, 2013) This type of exam has been used to assess learning all over the world, across all phases of education, ( Austin *et al.*, 2003; Khan *et al.*, 2013; Patricio *et al.*, 2013) and provides educators with the opportunity to more rigorously assess the effectiveness of their curricula on an ongoing basis ( Setyonugroho, Kennedy and Kropmans, 2015; Comert *et al.*, 2016; Kreptul and Thomas, 2016; Terry *et al.*, 2017).

Like many other CTSA awardees, the Michigan Institute for Clinical and Health Research (MICHR) has used the NCATS graduate level CTR competencies as a foundation for the development of its education and training programs ( Clinical & Translational Science Awards Program, 2019). In addition, MICHR also recognized the need to develop an objective assessment to measure students’ progression toward attaining program-defined competencies. To address this need, the Education and Mentoring Group at MICHR created a research OSCE (R-OSCE) to be used as one component of a comprehensive program to assess competence in specific NCATS-defined translational research domains. The exam was pilot-tested by pre-doctoral students early in their education, who were enrolled in a CTSA-sponsored introductory summer research immersion program to help evaluate their CTR training needs. Because limited information is available regarding use of an OSCE in this manner, the purpose of this work is to describe the development, feasibility, acceptability, and preliminary performance outcomes of the R-OSCE for use in a CTR training program.

## Methods

### Research OSCE development

This study was reviewed and judged to be exempt by the University of Michigan Health Sciences and Behavioral Sciences Institutional Review Board. As part of its CTSA mission to provide CTR training, MICHR created a 12-week summer research immersion program to introduce students with limited research experience in master’s degree and health professions degree programs to clinical and translational research. Students from our university, as well as from across the U.S. and Puerto Rico, are accepted into the program and engage in activities focused on interdisciplinary and collaborative work in translational and health disparities research ( Table 1).

**Table 1:** Participant Demographics

	2016 (n=18)	2017 (n=18)	2018 (n=20)
**Gender**			
Male Female	5 (28%) 13 (72%)	3 (17%) 15 (83%)	6 (30%) 14 (70%)
**Race**			
American Indian or Alaska Native Asian Black or African American More than one race Native Hawaiian or other Pacific Islander Other or not reported White	0 (0%) 3 (17%) 2 (11%) 2 (11%) 0 (0%) 1 (6%) 5 (28%)	2 (11%) 4 (22%) 4 (22%) 1 (6%) 0 (0%) 3 (17%) 4 (22%)	0 (0%) 3 (15%) 4 (20%) 1 (5%) 1 (5%) 2 (10%) 9 (45%)
**Ethnicity**			
Hispanic or Latinx	2 (11%)	1 (6%)	1 (5%)
**Stage of training**			
Professional degree (MD, PharmD, Dental, Nursing) PhD Master’s degree (MA, MS, MPH, MSW) Undergraduate degree (BA, BS) Not reported	8 (44%) 1 (6%) 9 (50%) 0 (0%) 0 (0%)	8 (44%) 0 (0%) 9 (50%) 0 (0%) 1 (6%)	11 (55%) 0 (0%) 5 (25%) 0 (0%) 4 (20%)
**Field of Study**	Biomedical Science (1) Kinesiology (1) Medicine (2) Pharmacy (6) Public Health (4) Social Work (4)	Dentistry (1) Engineering (1) Kinesiology (1) Medicine (2) Pharmacy (5) Public Health (4) Psychology (1) Social Work (2) Not reported (1)	Kinesiology (1) Medicine (4) Pharmacy (7) Public Health (4) Not reported (4)

Students work with a faculty mentor on an ongoing research project, as well as participate in a planned curriculum that explores substantive methodological and career topics related to clinical and translational research. The structured learning activities (e.g. activity-based seminars, journal club, group projects, community-based site visits, and reflective writing assignments) provide hands-on experience with a range of clinical and translational research activities and allow for consistent opportunities for interdisciplinary learning. The planned curriculum is aligned with seven of the 14 CTSA core thematic areas including: 1) clinical and translational research questions, 2) study design, 3) regulatory support and knowledge, 4) responsible conduct of research, 5) translational teamwork, 6) leadership, and 7) community engagement. Fifty-two competency statements are included in these seven thematic areas, and among those, 12 (23%) were identified as program competencies and used as the basis for developing the R-OSCE (see Supplementary File 1).

Faculty directors and staff who were knowledgeable about MICHR’s summer research immersion program and had experience developing OSCEs provided content for case studies that reflected these competency statements. Literature reviews were conducted to assist in creating appropriate content for the stations in contexts likely to be familiar to students. Sources included recently published clinical and translational research studies, recently published basic and preclinical research studies, reports prepared by government agencies and professional taskforces, and timely and relevant articles published by news outlets. The exam stations were reviewed by program instructional faculty and an instructional designer to ensure that all intended competencies were included in the exam and that the frequency of their sampling reflected the emphasis placed on the competency in the didactic portion of the curriculum.

Many students admitted to the summer research immersion program indicated they had no previous experience with an OSCE. Therefore, as part of their program orientation, students were given a shortened 5-station version of the R-OSCE as a practice test. This was intended to familiarize them with the format and content of an R-OSCE. Students completed a 7-station exam at the end of the 12-week program, which included a different set of stations from the practice test. The stations on both exams were designed to assess the same competencies, but they varied in the frequency of sampling (see Supplementary File 1).

Outstanding communication skills have been defined as one of the seven fundamental characteristics of a translational scientist ( Gilliland *et al.*, 2019). Written communication skills are crucial for a successful career in research, therefore a computer-based written format was chosen for the R-OSCE to help assess this critical skill. Research suggests that a computer-based format is an effective method for administering an OSCE ( Holyfield *et al.*, 2005). For the R-OSCE, this format allowed for ease of use for those taking the exam and allowed for fewer resources to implement it. The exam included case studies of situations students would be likely to encounter in a research setting as well as video vignettes depicting mentor/mentee interactions and informed consent encounters. Stations in both the pre-program and post-program exams contained data, figures, graphs, and relevant background material needed to successfully complete the specified tasks. Stations typically included two questions or tasks, although one station had one task and one station had four. This structure allowed for sampling of competencies across stations as well as an integrated application of multiple competencies within each station. This is realistic, as complex problems typically require researchers to apply knowledge and skills from different competency domains; and practical, as the exam was designed to be sufficiently comprehensive within the time-limits defined for its administration.

### Research OSCE Administration and Scoring

Students completed both the practice and end-program R-OSCEs in a private computer classroom setting. The exam was proctored by an instructional designer trained in OSCE development and administration. In advance of the exam, students were given instructions on the format, design, purpose, and duration of the R-OSCE. They were also informed that their performance would not impact their standing in the MICHR summer research immersion program. Students were also given the option to share their individual responses with their faculty mentors. Those who opted to share their responses received messages encouraging them to use the information when developing an individualized development plan with their faculty mentor, not only for their summer program experience, but for their future career development needs as well.

Students completed the exam using a secure, online survey platform (platform ( Qualtrics, 2019, https://www.qualtrics.com/). The exam stations were displayed in random order, ensuring that any given station’s score was not consistently biased by its relative order in the exam. Students advanced through the exam at their own pace, within a maximum 2.5-hour time limit. All students who completed the exams provided answers for all the stations; none were left blank.

Each R-OSCE was scored by multiple raters (between two and five), who received extensive training in the structure and use of a standardized scoring rubric. The scoring rubric was developed by the faculty and staff members who developed the R-OSCE, and was used to rate student responses for reasonable and appropriate content. Raters were trained to make judgments based on a) how well a student responded to the questions posed, b) how well their answers reflected an understanding of relevant issues, c) to what extent they demonstrated the ability to use data to respond to a question or perform a required task, d) their demonstration of critical thinking, and e) their overall performance on the station tasks. Numeric scores were assigned for each of the five criteria on a 1-5 scale (1=not demonstrated; 2=limited demonstration; 3=developing competence; 4=competent; 5=advanced), and average scores were calculated for each station. Once the scoring was complete, individual scores for both exams were sent to each participant (and their faculty mentors for those who gave consent) by secure email along with examples for each station demonstrating the elements of a ‘competent’ response.

## Results

The pre-program practice version of the R-OSCE was administered to 56 students in three cohorts of the summer research immersion program between 2016 and 2018 ( Table 1). The post-program exam was completed by 47 of these students. Students spent an average of 76 minutes completing the pre-program exam and 93 minutes completing the post program exam. Most students agreed to share their pre-program exam results with their mentors. The majority who completed the R-OSCE were female (75%) and currently enrolled in a professional degree or Master’s degree program (48% and 41% respectively). Students came from 21 universities and colleges and represented 9 disciplines. Most were completing degree programs in clinical fields such as medicine, pharmacy, or public health (68%), with the remainder completing degrees in social work, kinesiology, engineering, biomedical sciences, dentistry, psychology, and sports sciences.

The majority of students scored at the level of developing competence or better (3 or higher on a 1-5 scale) on most of the stations for the post-program exam ( Table 2). In each cohort, students generally performed well on the informed consent station, while a subset of students in each cohort struggled with stations associated with select competencies such as writing clinical and translational research questions and engaging communities in research.

**Table 2:** End program R-OSCE scores by station (Means and Standard Deviations)

Station	CTSA Core Thematic Area Assessed	2016(n=17)	2017(n=15)	2018(n=15)
1	Study Design Community Engagement	3.10 (.66)	2.90 (.89)	3.13 (.73)
2	Research Questions Scientific Communication	3.19 (.57)	2.28 (.79)	3.03 (.77)
3	Research Questions Translational Teamwork	3.09 (.38)	2.81 (.96)	2.78 (.58)
4	Responsible Conduct of Research Translational Teamwork	3.59 (.53)	3.72 (.82)	3.29 (.84)
5	Informed Consent	3.07 (.94)	4.66 (.33)	3.34 (.74)
6	Research Questions Study Design	3.13 (.50)	2.60 (.92)	3.00 (.71)
7	Scientific Communication Research Questions	3.21 (.67)	2.95 (.92)	3.13 (.59)

*Scores range from 1 to 5, where 1=Not Demonstrated, 2=Limited Demonstration, 3=Developing Competence, 4=Competent, 5=Advanced

### Feasibility and Acceptability

A subset of 20 students (2018 cohort) were asked to participate in a follow-up survey after completing the post-program OSCE in order to evaluate the acceptability of the assessment. Of the 15 students who completed the survey, only one-third reported having prior experience participating in an OSCE. Most (65%) agreed that the exam tasks were related to stated program competencies and that the stations presented realistic scenarios that could be encountered by clinical and translational researchers (80%). A majority (60%) also agreed that participating in the program helped them respond to the post program exam questions or tasks.

When asked what they liked most about the R-OSCE, students mentioned the computer-based format and the variety and diversity of the scenarios. They also liked being able to demonstrate what they had learned, being able to reflect on their past learning experiences, and thought the exam was a good way to get feedback on important skills. When asked what they liked least, students indicated that some of the case studies included too much information to process adequately, that they were unable to identify how the program seminars and/or their research project related to the content of the exam, or that the didactic curriculum did not prepare them for the exam.

## Discussion

The R-OSCE was designed to evaluate competency in specific CTSA-defined domains of clinical and translational research for students participating in a short-term introductory summer research immersion program. Findings from this pilot study show that it was possible and within our available resources to administer the computer-based R-OSCE to our students. By administering this exam, MICHR provided students with an opportunity to identify their strengths and gaps in select clinical and translational research knowledge, skills, and attitudes.

Allowing students to complete a practice R-OSCE before they began their training program enabled them to gain experience with the format and reflect on their current level of knowledge. The practice exam also had the potential to help students focus their attention on concepts and skills taught during the program curriculum. Enabling faculty mentors to see students’ practice test results may also have allowed them to use the information to develop individualized learning plans to address gaps in competency development. Finally, training program managers also benefitted from the R-OSCE, using the exam results to improve the curriculum for future cohorts. For example, results from the R-OSCE suggested that some students struggled with writing clinical and translational research questions and understanding how to engage communities in research. Thus, the curriculum was refined to pay more attention to the instruction and application of these critical research skills.

Although most students scored at the level of developing competence for most of the R-OSCE stations, some students did have lower scores. Given that this program was specifically designed for students with limited research experience, as well as the diversity of the program cohorts, each student came into the program with a different educational background and level of prior research knowledge. Also, while some competencies were sampled multiple times on the OCSE, (e.g. “writing clinical and translational research questions” was sampled seven times), other competencies were sampled less often (e.g. “building a translational team and study design” was sampled two times). Thus, context specificity may have played a role in student performance on stations that assessed the less frequently sampled competencies. Also, because the R-OSCE was a low-stakes exam (i.e., students were informed that their OSCE scores would not affect their participation in the program), students may not have felt motivated to perform their best which may have resulted in lower scores. Future revisions to the content and design of the stations that take student diversity and context specificity into account may increase student engagement with the exam.

### Limitations

There were several limitations to this study. First, while OSCEs are considered to be a gold standard of competency assessment, they require significant resources and can be costly to develop, administer, and score ( Zartman *et al.*, 2002; Kelly and Murphy, 2004; Holyfield *et al.*, 2005; Patricio *et al.*, 2013; Abdelaziz *et al.*, 2016; Ogunyemi and Dupras, 2017). Due to resource limitations, the R-OSCE was developed to fit a modest budget and limited resources. Second, although a computer-based format proved useful in reducing the administrative burden of the exam, it did not allow us to directly assess students’ interpersonal skills and may not have been an accurate reflection of an individual’s skill ( Holyfield *et al.*, 2005). The addition of virtual standardized patients to the OSCE would permit future researchers to directly assess communication skills, however it would necessitate additional cost and resources to implement. Despite this, scores for the R-OSCE indicated clear learning needs for students, which was beneficial for them and their faculty mentors as they developed individualized learning plans.

Finally, identifying, training, and retaining raters for the R-OSCE required substantial time and resources. Despite measures taken to ease the burden on raters, the time needed to score the exams was considerable. Revisions to the scoring rubric and use of a consistent cohort of raters may decrease this burden in the future.

## Conclusion

This work represents a new application of the OSCE, which is a well-accepted method for the assessment of skills development in medical education. The online administration format can facilitate access to the exam, providing a solution to the current need for remote access to assessments ( Boursicot *et al.*, 2020). Results show that the R-OSCE was well-received by the students and was feasible to administer. This exam is an innovative assessment that, in combination with other rigorously designed and validated instruments, could provide information to students and training program administrators to determine whether learners have acquired relevant research competencies. We believe that our findings will be of interest to administrators of research education programs for students in graduate and professional degree programs as well as those in trainee, translational science PhD programs and fellowship programs.

## Take Home Messages

• The R-OSCE was shown to be feasible to administer to students in a clinical and translational summer research program.
• Most students performed across the range from “not demonstrated” (1) to “competent” (4); in a few cases, students performed at an “advanced” (5) level.
• Once validated, the R-OSCE can assist in identification of student learning needs and also inform curriculum planning.
• Most of the students had a favorable opinion of the R-OSCE.
• Further studies are needed to validate the use of the R-OSCE for formative and summative assessment of competency in clinical and translational research.

## Notes On Contributors

All of the co-authors contributed to the development of this work, informed the conclusions it advances, and participated in all rounds of revisions required for submission.

Phillip A. Ianni PhD, is a Program Evaluation Specialist at the Michigan Institute for Clinical and Health Research at the University of Michigan. ORCID ID: https://orcid.org/0000-0003-1264-7322

Brenda L. Eakin MS, is the Program Director for Career Development and Mentoring Education at the Michigan Institute for Clinical and Health Research at the University of Michigan. ORCID ID: https://orcid.org/0000-0002-7972-8621

Elias M. Samuels PhD, is the Program Director for Evaluation and Workforce Development at the Michigan Institute for Clinical and Health Research at the University of Michigan. ORCID ID: https://orcid.org/0000-0002-6725-3382

Ellen Champagne BA, is an Evaluation Project Manager at the Michigan Institute for Clinical and Health Research at the University of Michigan.

Vicki L. Ellingrod, PharmD, FCCP is Associate Director at the Michigan Institute for Clinical and Health Research and Associate Dean for Research and John Gideon Searle Professor of Clinical and Translational Pharmacy, College of Pharmacy at the University of Michigan. ORCID ID: https://orcid.org/0000-0001-8289-4834
