# Supplementary material for: The Research Objective Structured Clinical Exam (R-OSCE): An Innovative Tool to Assess Clinical and Translational Research Competencies
Source: MedEdPublish (2016). 2021 Sep 3;10:143. Originally published 2021 May 24. [Version 2] doi: 10.15694/mep.2021.000143.2 (PMC10939553; doi:10.15694/mep.2021.000143.2)
Supplement: Peer review history

# Reviewer report: Reviewer response for version 2

**DOI:** 10.21956/mep.20215.r31300

**Published:** 2021-09-15

**Authors:** Julie Hunt

**Affiliations:** 1 Lincoln Memorial University

This review has been migrated. The reviewer awarded 4 stars out of 5 The authors describe an examination to assess competency in clinical and translational research. The exam given was demonstrated to be feasible and well accepted by participants and educators. However, I disagree that the exam was truly an OSCE, which is a multi-station exam with a different rater for each station (this is important for generating reliable, valid results), and requires the participant to be observed while performing a task. I commend the authors for the use of a pre-test, as I think this practice was likely helpful to students, and there is a learning curve for students when first encountering a new exam type or process. The authors describe using multiple raters but do not mention a measure of inter-rater reliability. Inter-rater reliability could have been calculated if students, or a subset of students, were evaluated by more than one rater. The inclusion of an inter-rater reliability measure would have supported the reliability and validity of the exam format.

Reviewer Expertise:

NA

No decision status is available

# Reviewer report: Reviewer response for version 2

**DOI:** 10.21956/mep.20215.r31301

**Published:** 2021-09-07

**Authors:** BALAJI ARUMUGAM

**Affiliations:** 1 TAGORE MEDICAL COLLEGE AND HOSPITAL

This review has been migrated. The reviewer awarded 3 stars out of 5 Thanks for the wonderful way of conduct of the study and special thanks for the opportunity to review this article which speaks on new educational tool. The R-OSCE was designed to evaluate competency in specific CTSA-defined domains of clinical and translational research for students participating in a short-term introductory summer research immersion program. Most students performed across the range from “not demonstrated” (1) to “competent” (4); in a few cases, students performed at an “advanced" (5) level. The internal validity of the study would a have been affected by validation process of OSCE and the interdisciplinary students with varied level of exposure in research. The study was well conducted in spite of several limitations.

Reviewer Expertise:

NA

No decision status is available

# Reviewer report: Reviewer response for version 1

**DOI:** 10.21956/mep.19148.r27690

**Published:** 2021-07-12

**Authors:** Trevor Gibbs

**Affiliations:** 1 AMEE

This review has been migrated. The reviewer awarded 4 stars out of 5 I enjoyed reading this paper and thought that it showed a very innovative approach to the research although as pointed out by the authors, there are several limitations. I applaud the development of the competencies in the area.I found it difficult to see why the authors compared this to the OSCE, calling it a research OSCE. The OSCE was designed for a specific function and I felt that this approach was not following those guidelines and could have been held as an online assessment approach.Despite my reservations I would recommend this paper to those involved in clinical research and its assessment.

Reviewer Expertise:

NA

No decision status is available

# Reviewer report: Reviewer response for version 1

**DOI:** 10.21956/mep.19148.r27689

**Published:** 2021-05-31

**Authors:** BALAJI ARUMUGAM

**Affiliations:** 1 TAGORE MEDICAL COLLEGE AND HOSPITAL

This review has been migrated. The reviewer awarded 3 stars out of 5 Thanks for the invite to review this article. The study was done among the participants who underwent 12 week program on research and the outcome was assessed based on scoring obtained in OSCE as the assessment tool both during and post program. The article was well written with clear objectives and the methodology. Kudos to the authors for using computer based OSCE as the assessment tool for a research program. As a reviewer out of curiosity, I wan to know little more information on the article. Thanks Queries are 1. Was the assessment method announced to the study participants before the start of the prog?2. Do the study participants know that they are under a research study for future publication? 3. What was the time interval between the administration of the OSCE after the end of 12 week program?4. Please explain little more on the administration of OSCE? Was the OSCE done completely online (means through a web link)? Did the 12 week Summer research immersion program conducted by offline in classroom or was it an online program? 5. Did the listed competencies were broken into SLOs and mentioned to the study participants before or during the prog by the faculty?

Reviewer Expertise:

NA

No decision status is available
